# The Overlooked Burden of Atopic Comorbidities in Eosinophilic Esophagitis: Insights from a Real-Life Comprehensive Multidisciplinary Evaluation

**DOI:** 10.3390/jcm14207322

**Published:** 2025-10-16

**Authors:** Marcella Pesce, Mario Ricchiuti, Elena Cantone, Maddalena Napolitano, Aikaterini Detoraki, Michele Falco, Pierpaolo De Giorgi, Roberto Berni Canani, Mauro Maniscalco, Giovanni Sarnelli

**Affiliations:** 1 Department of Clinical Medicine and Surgery, University of Naples “Federico II”, Via Pansini 5, 80131 Naples, Italy; marcella.pesce@unina.it (M.P.); mario.ricchiuti@unina.it (M.R.); maddalena.napolitano@unina.it (M.N.); michele.falco@unina.it (M.F.); pierpaolo.degiorgi@unina.it (P.D.G.); mauro.maniscalco@unina.it (M.M.); 2 Department of Neuroscience, Reproductive Sciences and Dentistry, University of Naples “Federico II”, 80131 Naples, Italy; elena.cantone@unina.it; 3 Department of Internal Medicine and Clinical Complexities, A.O.U. Federico II, 80131 Naples, Italy; kate.detoraki@gmail.com; 4 ImmunoNutritionLab and NutriTechLab, CEINGE Advanced Biotechnologies and Department of Translational Medical Science, University of Naples Federico II, 80131 Naples, Italy; roberto.berni@unina.it

**Keywords:** Eosinophilic esophagitis, allergic comorbidities, type 2, multidisciplinary approach, real-life, atopic march

## Abstract

**Background/Objectives:** Eosinophilic esophagitis (EoE) is a T2-mediated disease characterized by dysphagia and food impaction. It is often associated with other atopic disorders and is considered a late manifestation of the “atopic march”. In clinical practice, allergic comorbidities are frequently underdiagnosed and primarily based on self-reporting, potentially underestimating the true burden of T2-related pathology. To address this, a multidisciplinary task force was established at our tertiary center to systematically evaluate newly diagnosed patients with EoE. **Methods**: This cross-sectional observational study included patients referred for EoE evaluation from January 2022. Clinical history was collected prospectively, with systematic assessment for T2 comorbidities. All patients underwent an esophagogastroduodenoscopy with esophageal biopsies. Following EoE diagnosis, patients were referred to dermatology, ENT, immunology, and respiratory specialists. **Results**: A total of 43 patients were enrolled. Anamnestic T2 comorbidities were reported by 88% of patients. Rhinitis was the most common, while at baseline, no patients reported chronic rhinosinusitis with nasal polyps (CRSwNP). After specialist evaluation, diagnoses of asthma, food allergy, and atopic dermatitis remained stable, while eight patients previously reporting rhinitis were newly diagnosed with CRSwNP. Overall, 65% of patients had ≥2 T2 comorbidities in addition to EoE, and 25% had ≥3. **Conclusions**: Our findings support a multidisciplinary approach to assess T2 comorbidities in patients with EoE, with a high overall prevalence (95.3%) and frequent coexistence of multiple atopic conditions. CRSwNP was frequently underdiagnosed and only identified after rhinofibroscopy. Although our data needs to be confirmed in larger multicenter studies, our results suggest that relying solely on patient-reported history or single-specialty evaluation risks underestimating the systemic nature of the T2 inflammatory pathway in EoE.

## 1. Introduction

Eosinophilic esophagitis (EoE) is a chronic, immune-mediated inflammatory disease of the esophagus, defined by symptoms of esophageal dysfunction, such as dysphagia and food impaction, and histological evidence of eosinophilic inflammation, in the absence of other causes of esophageal eosinophilia [1,2]. EoE is recognized as a type 2 (T2)-mediated disease, driven by an aberrant adaptive immune response, which promotes eosinophil recruitment, activation, and survival, leading to inflammation and tissue remodeling [3,4,5]. The common immunologic profile underlines the strong association between EoE and other T2 comorbidities, including allergic rhinitis, chronic rhinosinusitis with nasal polyps (CRSwNP), asthma, and atopic dermatitis, supporting the hypothesis that EoE represents a late manifestation of the so-called “atopic march” [6,7]. Indeed, previous data strongly supports this overlap, demonstrating that up to 80% of patients with EoE have at least one atopic comorbidity, including allergic rhinitis (approximately 60%), asthma (30–40%), or food allergies (20–40%) in population-based studies [8,9,10]. Moreover, emerging evidence suggests that the presence and type of T2 comorbidities may affect some EoE features, including the number of eosinophils in the mucosa, possibly leading to fibrotic complications [11,12]. Despite this well-known association, the routine clinical management of patients with EoE often falls short in addressing the full spectrum of T2-related pathology due to limitations in traditional diagnostic paradigms. Recently, Schoepfer et al. [13] collected real-world data about the prevalence of T2 comorbidities reported by physicians in a large pediatric and adult EoE population. In this study, T2 comorbidities were recognized by physicians in only 45% of the overall adult population, with a reported prevalence of 20% for both asthma and rhinitis. Notably, less commonly reported conditions, such as CRSwNP, were not captured in this study, suggesting that these may be underdiagnosed in routine care. In fact, clinical evaluation is frequently based on patient-reported history of atopic comorbidities, which can increase the risk of underdiagnosing and/or misclassifying associated T2 conditions. Furthermore, a recent paper by Redd et al. [12] outlined that despite the similar response rate to topical corticosteroids, patients with EoE with multiple atopic comorbidities showed significant differences in disease presentation, with a substantially different symptom profile, more exudates and edema at baseline endoscopy, and higher peak eosinophil counts at histology. Current guidelines recognize that a multidisciplinary team approach is pivotal to properly diagnosing and managing patients with EoE, since comorbid patients may benefit from a unified management plan that could influence the choice of treatment [14,15]. Despite the growing recognition of the burden of atopic diseases in EoE, referral criteria to guide inter-consultation between specialists are currently lacking. As EoE is a late phase of the atopic march, most patients with EoE may already be receiving treatment for a comorbid T2 disorder. On one hand, specialists’ consultations might be considered unnecessary for patients with well-controlled T2-related disorders who do not require additional therapy, as it could considerably increase healthcare costs without affecting clinical decision-making. On the other hand, patients may not reliably report T2-related symptoms or comorbidities that could inform more tailored therapeutic strategies and lead to personalized care in patients with EoE. The primary aim of this study was to evaluate the effectiveness of a multidisciplinary task force in systematically identifying T2-related comorbidities in patients with EoE. By integrating expertise from dermatology, ENT, immunology, and pulmonology, this approach sought to enhance diagnostic accuracy, uncover previously underdiagnosed atopic conditions, and improve our understanding of the overlapping pathophysiology of T2 disorders. By systematically evaluating the burden of T2-mediated diseases, we also aimed to investigate their potential impact on EoE clinical presentation and response to therapy, to provide these patients with more comprehensive and personalized care.

## 2. Materials and Methods

### 2.1. Study Design

This was a cross-sectional, observational study conducted at a tertiary referral center, involving consecutive patients with suspected EoE referred from January 2022. Patients were prospectively enrolled and were included in the study if they had suggestive symptoms of EoE, such as dysphagia or food impaction, and received a histological confirmation of the disease. According to most recent EoE guidelines, all patients underwent esophagogastroduodenoscopy (EGD) with differential esophageal biopsies from at least two anatomical regions between proximal, mid, and distal esophagus. Histopathological examination was performed to confirm the diagnosis of EoE, defined by the presence of ≥15 eosinophils per high-power field in esophageal tissue, in the absence of alternative causes of esophageal eosinophilia [16]. EoE Endoscopic Reference Score (EREFS; Edema, Rings, Exudates, Furrows, and Strictures) was also evaluated in all patients during endoscopy [17]. At baseline, no patient was receiving any EoE-specific treatment. A total of 23 patients (54%) had already been treated with PPIs prior to enrollment after the onset of esophageal symptoms or following a food impaction event, but all patients were required to stop the medication prior baseline endoscopy for at least 4 weeks.

Detailed clinical histories were obtained from all patients, focusing on the onset and progression of EoE symptoms, as well as past and current allergic or atopic conditions. Patients were specifically questioned about their history of asthma, allergic rhinitis, food allergies, atopic dermatitis, and other T2-mediated conditions.

The clinical severity of EoE symptoms was assessed through the standardized Eosinophilic Esophagitis Symptom Activity Index (EEsAI), a validated score using Patient-Reported Outcomes (PRO) items evaluating the characteristics of dysphagia, behavioral adaptations to living with dysphagia, and pain while swallowing over a 7-day recall period. The PRO instrument consisted of 45 items. The domain addressing symptoms while eating or drinking includes items on duration, frequency, and severity of dysphagia, time required for meal intake, dysphagia upon consuming liquids, and pain when swallowing. The Visual Dysphagia Question subitem (VDQ) addresses the severity of dysphagia when consuming food of 8 distinct consistencies. The behavioral adaptations [avoidance, modification, and slow eating (AMS) of various foods] is assessed in the context of consuming the same food consistencies [18]. The prevalence and severity of GERD symptoms were investigated using the GERDQ score, a validated score based on 6 questions establishing the frequency of heartburn, acid regurgitation, epigastric pain, and nausea, also evaluating the need for using antacid medications and the presence of nocturnal symptoms [19].

Patients with EoE were assigned to first-line therapeutic approaches while investigating concomitant T2-disease through specialists’ referral. Patients were considered responders to treatment if the peak eosinophilic count in the esophageal mucosa was reduced below <6 eos/HPF in all biopsies at a 12-week follow-up endoscopy [15,20]. Informed consent was obtained from all patients.

### 2.2. Specialist Consultation

Following histological confirmation of EoE, patients were systematically referred for comprehensive multidisciplinary evaluation. For all patients, consultations were performed by the same expert specialist. No blinding was applied given the real-life, multidisciplinary design. This included:

#### 2.2.1. Dermatology Consultation

Medical history and physical examination to assess the extent and localization of atopic dermatitis, as well as any associated cutaneous outcomes, using both objective and subjective clinical scoring to assign numerical values that aid in treatment decision-making and therapeutic follow-up [21].

#### 2.2.2. ENT Evaluation

Rhinofibroscopy was performed to identify chronic rhinosinusitis with nasal polyps (CRSwNP) or to confirm allergic rhinitis. CRSwNP was diagnosed based on rhinofibroscopy findings, supported by patient history and symptomatology [22,23].

#### 2.2.3. Immunology and Allergy Assessment

Skin prick tests and serum specific IgE were conducted, as they were necessary to confirm sensitization to environmental or food allergens. Allergic rhinitis and food allergies were diagnosed according to ARIA (Allergic Rhinitis and its Impact on Asthma) and EAACI (European Academy of Allergy and Clinical Immunology) guidelines [24,25].

#### 2.2.4. Pulmonology Evaluation

Asthma was evaluated through clinical evaluation and spirometry. Asthma diagnosis was confirmed based on GINA (Global Initiative for Asthma) criteria, which include the presence of reversible airflow limitation and a history of respiratory symptoms such as wheezing, shortness of breath, or cough [26].

### 2.3. Statistical Analysis

This cross-sectional study was conceived as an exploratory, descriptive assessment of comorbidities in EoE within a multidisciplinary setting. Accordingly, we did not perform an a priori power calculation; the sample size corresponds to all consecutive eligible patients seen during the predefined enrollment period, reflecting feasibility in a relatively uncommon disease and the resource requirements of comprehensive phenotyping. Data were analyzed to determine the prevalence of T2-related comorbidities and to assess the concordance between patient-reported histories and specialist-confirmed diagnoses. Categorical variables were summarized as frequencies and percentages, and continuous variables were expressed as median (interquartile range, IQR). Comparisons between patient-reported and physician-confirmed diagnoses were performed using McNemar test for paired data. Comparison between continuous variables were performed with T-test for unpaired data after confirming normal distribution of the variables. For variables not following normal distribution, Mann–Whitney U test for unpaired data was performed. A *p*-value < 0.05 was considered statistically significant.

## 3. Results

### 3.1. Study Population

A total of 43 patients with EoE were prospectively enrolled, including 35 males (70%) with a median age of 26 (21.5–35) years. The demographic and clinical characteristics of the study population are summarized in Table 1.

Overall, T2 comorbidities were reported by 88% of the cohort (38 patients). Allergic rhinitis was the most reported comorbidity (76.7%, 33 patients), while no patients reported CRSwNP at baseline. Asthma was reported by 17 patients (39.5%), atopic dermatitis (AD) was noted in 8 (18.6%), and food allergies in 14 (32.5%). Prior to specialist consultation, 20 patients (46.5%) reported ≥2 T2 comorbidities, and seven (16.3%) reported ≥3.

### 3.2. Post-Consultation Analysis of T2 Comorbidities

Specialist consultations led to the identification of T2 comorbidities in 41 patients (95.3%), with three additional patients diagnosed with previously unreported T2 diseases. Although not statistically significant, a slight increase in IgE-mediated food allergy prevalence (32.5% to 44.1%) was observed. Conversely, asthma prevalence decreased (39.5% to 32%) due to spirometry-based exclusion of three patients. Similarly, AD prevalence declined (18.6% to 13%) after one patient was reclassified with dermographic urticaria (all *p* = ns). In contrast, after ENT evaluations, we observed a significant increase in CRSwNP prevalence (0% to 18.6%, *p* = 0.01), since nearly 20% of the overall population complained of chronic symptoms of nasal obstruction but were unaware of the diagnosis of CRSwNP. We also noted a general rise in rhinitis prevalence (76.5% to 90.6%, *p* = ns), though not statistically significant.

**Figure 1:**
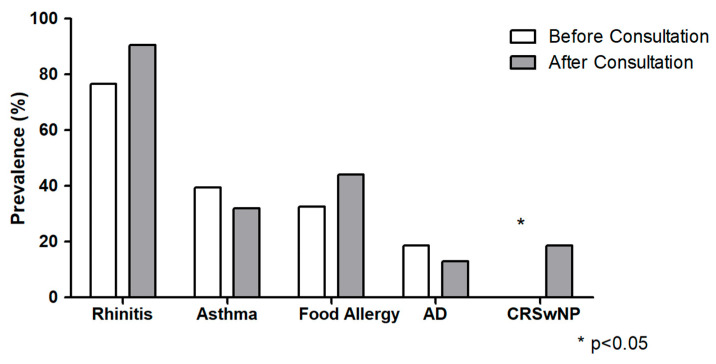
Prevalence of T2 comorbidities before and after specialist consultation. Atopic dermatitis (AD); chronic rhinosinusitis with nasal polyps (CRSwNP).

Post-consultation, the number of patients with ≥2 T2 comorbidities significantly increased (65.1% vs. 46.5% *p* = 0.01), substantiating the risk of underestimating the T2-related burden in this population of patients. Overall, 65% of the whole population had two or more concomitant atopic disorders aside from EoE and 10 patients (23.2%) had three or more. The prevalence of T2 comorbidities is summarized in Figure 1.

Patients with ≥2 T2 comorbidities showed no significant differences in age at diagnosis [25 (20.75–31.75) (95% Confidence Interval (CI), 24.26–32.59) vs. 29 (22.5–44.5) (95% CI, 25.86–41.87) years), age of onset [17 (9.5–28.3) (95% CI, 14.87–25.70) vs. 20 (12–35.5) (95% CI, 14.52–33.88) years], diagnostic delay [7 (3.75–12.25) (95% CI, 5.77–10.50) vs. 6 (2–13.5) (95% CI, 4.42–14.91) years], or eosinophil counts [350 (240–545) (95% CI, 238.78–781.21) vs. 330 (240–510) (95% CI, 292.35–520.98) cells/µL]. Also, both EREFS scores and the prevalence of esophageal strictures were comparable between the two populations of patients [3 (2–4) (95% CI, 2.28–3.50) vs. 3 (1–4) (95% CI, 1.83–3.49) and 21.4% vs. 6.6%, respectively; both *p* = ns]. On the other hand, we observed that patients with ≥2 comorbidities reported significantly fewer episodes of food impaction requiring emergency admission (66.7% vs. 28.6%, *p* < 0.05). The differences between the number of comorbidities before and after specialist consultations are portrayed in Supplementary Figure S1.

No significant differences emerged in total EEsAI score [27 (12–37.25) (95% CI, 18.22–36.35) vs. 12 (0–36) (95% CI, 8.85–35.41)] and in both VDQ and AMS subscores [2.04 (0.75–3.5) (95% CI, 1.53–3.10) vs. 0.83 (0–2.7) (95% CI, 0.41–3.63) and 2 (0.4–4.6) (95% CI, 1.60–3.62) vs. 0.86 (0–2.7) (95% CI, 0.55–1.95); all *p* = ns].

Interestingly, patients with fewer T2 comorbidities had higher GERDQ scores [9.5 (8.75–12.25) vs. 7 (6.5–10), *p* < 0.05] as compared to comorbid patients.

### 3.3. Impact of Specific T2 Comorbidities on Disease Phenotype and Therapeutic Response

CRSwNP-diagnosed patients did not differ significantly in age [26 (21.5–31.5) (95% CI, 20.79–33.71) vs. 26.5 (21.75–37.25) (95% CI, 26.53–36) years], age at onset [15.5 (8–24.5) (95% CI, 8.27–27.72) vs. 19 (10.75–31.25) (95% CI, 17.01–28) years], or diagnostic delay [8.5 (4.75–13) (95% CI, 3.89–14.60) vs. 6.5 (2–11.5) (95% CI, 5.91–11) years]. EREFS score [3 (2–3.25) (95% CI, 1.83–3.92) vs. 2.5 (1–3.25) (95% CI, 2.11–3)], stricture prevalence (12.5% vs. 17.1%), and history of food impaction (25% vs. 46%) were also similar. However, CRSwNP patients showed significantly higher EEsAI scores [34 (29.25–55.25) vs. 15 (0–34.5); *p* < 0.05) and AMS subscore [4.5 (1.81–7.23) vs. 1.1 (0–2.6); *p* < 0.05], mirroring an increased number of adaptive behaviors to cope with dysphagia in this population of patients. In contrast, the VDQ subscore, although increased, failed to reach statistical significance [3.09 (1.9–4.7) (95% CI, 1.58–5.23) vs. 1.02 (0–2.6) (95% CI, 1.14–3); *p* = ns].

**Figure 2:**
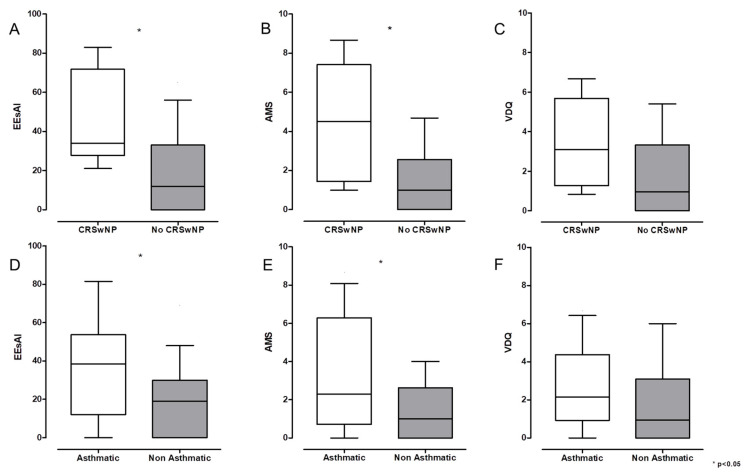
Dysphagia burden and behavioral adaptations in patients with EoE with concomitant CRSwNP (**A**–**C**) and asthma (**D**–**F**). Both total eosinophilic esophagitis symptom activity index (EEsAI) (**A**,**D**) and avoidance, modification, and slow eating (AMS) (**B**,**E**) subitem were significantly higher in comorbid patients with CRSwNP and asthma, while the visual dysphagia question (VDQ) (**C**,**F**) subitem failed to reach significance in both populations of patients. Chronic rhinosinusitis with nasal polyps (CRSwNP). Data are expressed as median ± IQR.

Accordingly, patients with asthma had significantly higher total EEsAI [38.5 (14.25–49.75) vs. 19 (0–27); *p* < 0.05] and AMS scores [2.29 (0.86–6) vs. 1 (0–2.57); *p* < 0.05], while VDQ did not significantly differ [2.15 (0.99–4.17) (95% CI, 1.52–4.08) vs. 0.95 (0–2.86) (95% CI, 1.02–2.83); *p* = ns]. No differences were observed in age [29 (22.25–35.5) (95% CI, 23.97–37.18) vs. 25 (21–32) (95% CI, 25.31–35.09) years], age of onset [15.5 (8.75–33.5) (95% CI, 13.21–30.03) vs. 18 (10–25) (95% CI, 15.59–27.30) years], diagnostic delay [6.5 (2.5–14.25) (95% CI, 4.39–12.60) vs. 7 (3–12) (95% CI, 5.86–11.65) years], EREFS scores [3 (2–4) (95% CI, 2.07–3.93) vs. 2 (1–3) (95% CI, 2.01–2.96)], stricture prevalence (28.6% vs. 10.3%), food impaction history (21.4% vs. 51.7%) or GERDQ score [7 (6.25–9.5) (95% CI, 6.34–9.06) vs. 9 (7–12) (95% CI, 8.34–10.52); all *p* = ns]. No significant differences were observed for all the other T2 comorbidities analyzed. The symptoms score of comorbid patients with CRSwNP and asthma are depicted in Figure 2.

After first-line pharmacological treatment, 33 patients (77%) achieved histological remission, 17 (51.5%) on PPI, and 16 (48.5%) on topical swallowed corticosteroids. No significant association was observed between the presence and type of T2 comorbidities and the response rate to either PPI or budesonide.

## 4. Discussion

The study highlights the significant burden of type 2 inflammatory comorbidities in adult patients with EoE, reinforcing the knowledge that EoE rarely occurs as an isolated entity. Indeed, nearly 90% of our studied cohort reported at least one allergic comorbidity, indicating that both demographic and clinical characteristics of our population are broadly consistent with those observed in larger national or European cohorts [8]. A structured, multidisciplinary consultation increased this detection rate even further, by uncovering CRSwNP in nearly one in five patients.

To date, the REVEAL study by Brailean et al. [27] is the only published paper reporting the prevalence of CRSwNP among patients with EoE, identifying a rate of 12.0% in a large US population. In our cohort, CRSwNP was present in 18.6% of patients with EoE, a notably higher proportion. Even considering the evidence from a recent review that the incidence and prevalence of EoE is heterogenous, reflecting differences in population features such as geographical location, ethnicity, gender, and age [28], our data reinforces the relevance of shared type 2 inflammatory pathways. The lack of data on this association in the literature underscores the importance of our findings and highlights the need for further prospective studies exploring the clinical impact of CRSwNP in EoE. This result is even more noticeable if considering that at baseline, none of the patients were aware of this comorbid disorder since they routinely complained of symptoms of chronic nasal obstruction and anosmia that had never been properly investigated. Although asthma and rhinitis were the most reported T2 diseases, CRSwNP stood out for its clinical implications. Patients with a new diagnosis of CRSwNP had a significantly higher symptom burden, particularly in behavioral adaptation to dysphagia. Prior studies in CRSwNP cohorts (outside the EoE context) consistently report greater symptom burden, reduced quality of life, and increased functional limitations compared to non-CRSwNP controls [29]. However, to our knowledge, no study has specifically evaluated patients of EoE with comorbid CRSwNP in terms of esophageal symptoms and EoE-specific adaptive behaviors (e.g., food modification, eating pace, avoidance). Although further larger multicenter studies are needed to confirm our findings, these results suggest that the presence of nasal polyps may not be just an epiphenomenon but could reflect a more systemic, severe endotype of T2 inflammation affecting both the upper airway tract and the esophagus. Importantly, the detection of additional comorbidities post-consultation consistently altered clinical stratification [30], with a significant increase in patients classified as having two or more atopic conditions. This suggests that patient-reported history or single-specialty evaluation carries the risk of underestimating the systemic nature of the T2 inflammatory pathway in EoE. Indeed, in a recent study by Schoepfer et al. [13], physicians recognized T2 comorbidities in only 45% of the overall adult population. Notably, the physician-reported prevalence of CRSwNP was not included, suggesting that this specific type of T2 comorbidity may be greatly underdiagnosed in routine clinical practice.

On the contrary, patients with fewer than two comorbidities reported more food impactions and higher GERD-related symptom scores. Despite further studies, using objective reflux testing would be needed to clearly identify the role of GERD in patients with EoE with varying comorbidity profiles; this result is of interest since it could lead to speculation that in patients with no atopic comorbidity, the main pathogenic drive of the disorder could be linked to the coexistence of GERD-related symptoms, which are more prevalent as compared to patients with multiple allergic comorbidities.

Nonetheless, whether this reflects differences in disease phenotype, age distribution, or can be related to symptom misattribution or diagnostic overlap, remains an open question and further larger prospective studies are required to coherently interpret these findings.

Consistently with Redd et al. [12], our patients with multiple atopic comorbidities responded similarly to first-line therapies compared to patients with less than two. In fact, we observed no significant difference in treatment outcomes to both oral budesonide and high-dose proton pump inhibitors, regardless of the prevalence and type of T2 comorbidity, with comparable rates of histologic remission. This result confirms the efficacy of first-line therapies, even in the presence of multiple T2 comorbidities.

Despite similar response rates to first-line therapies, the presence of multiple T2 inflammatory disorders with shared pathogenic pathways may profoundly alter clinical decision-making, not only from the gastroenterological perspective. For instance, Sarhan et al. [31] demonstrated that among patients undergoing surgery for CRSwNP, those with comorbid EoE experienced a 100% recurrence rate of nasal polyps within a one-year follow-up, underscoring the need to tailor management strategies in this subgroup.

While our findings support the value of multidisciplinary assessment in improving the detection of T2 comorbidities in EoE, the balance between clinical benefit, healthcare resource utilization/costs, and feasibility of this approach warrants further consideration. Multidisciplinary care may lead to earlier diagnosis and more effective management of associated atopic conditions, potentially reducing long-term morbidity, increasing patients’ satisfaction and reducing the healthcare burden. However, such an approach may not be adaptable to all clinical settings due to limitations in infrastructure, specialist availability, and costs. Moving forward, a stepwise referral strategy based on significant clinical stratification of the patients could improve feasibility and optimize resource allocation. Existing literature in both eosinophilic gastrointestinal disorders and other chronic T2 diseases suggests that integrated care models can improve diagnostic efficiency and patient satisfaction and may ultimately prove cost-effective by reducing unnecessary investigations and treatment delays [32]. Nevertheless, further research is needed to evaluate the long-term health-economic impact of multidisciplinary care pathways in EoE.

## 5. Conclusions

This study has several limitations. Firstly, it was conducted at a single tertiary care center with a relatively limited sample size, which may hamper the transferability of our findings to broader populations and limit the strength of statistical association, especially on subgroups analysis. Secondly, the cross-sectional design does not allow for conclusions regarding causality between T2 comorbidities and symptom burden. Furthermore, detailed data regarding ongoing treatments for atopic conditions at baseline were not systematically collected in our cohort, potentially interfering with the overall symptoms burden and the detection of comorbidities. Finally, we did not evaluate longitudinal outcomes following the multidisciplinary assessment, so its impact on long-term disease management, and potential effects of a single therapy for multiple T2 comorbidities remains to be established.

Despite these limitations, our results clearly support the need for a multidisciplinary team approach to investigate the burden of T2-related pathology in paper with EoE. In our hands, the prevalence of T2-related comorbidities was as high as 95% of patients with EoE and up to 65% of our population had two or more allergic disorders. Moreover, CRSwNP is frequently underdiagnosed and properly identified only following rhinofibroscopy. This is highly relevant when managing patients with EoE because it underlines how targeting shared pathogenetic pathways can lead to improved therapeutic outcomes, guiding the most appropriate choice of therapy. Multidisciplinary evaluation uncovers diagnoses that would otherwise be missed, and these comorbidities, particularly CRSwNP and asthma, are associated with a different symptom burden and distinct behavioral adaptations. As seen in our study, a multidisciplinary approach significantly enhances the detection and management of T2-related comorbidities in patients with EoE.

By uncovering underdiagnosed conditions, like CRSwNP, and refining self-reported histories, this strategy offers a more comprehensive framework for managing EoE and its associated atopic disorders. Going forward, larger prospective studies will be essential to clarify how systemic allergic inflammation shapes the course of EoE, and how to best intervene.

## Figures and Tables

**Table 1:** Baseline demographics, clinical characteristics and T2 comorbidities of the study population (*n* = 43). Data are given as *n* (%) and median (IQR).

Demographics	
Sex, M (%)	35 (81.4%)
Age (years)	26 (21.5–35)
Age at symptom onset (years)	17 (10–29.5)
Diagnostic delay (years)	7 (2.5–12.5)
Peripheral blood eosinophil count (cells/μL)	330 (240–510)
Endoscopic features	
EREFS score	3 (1.5–4)
Esophageal strictures, *n* (%)	7 (16.3%)
Endoscopic dilation, *n* (%)	2 (4.7%)
Food impaction requiring emergency care, *n* (%)	18 (41.9%)
Symptoms scores	
EEsAI score	21 (6–36.5)
VDQ subscore	1.9 (0.24–3.33)
AMS subscore	1.25 (0–2.83)
GERDQ score	9 (7–10)

## Data Availability

The raw data supporting the conclusions of this article will be made available on request from the corresponding author.

## Supplementary Materials

The following supporting information can be downloaded at: https://www.mdpi.com/article/10.3390/jcm14207322/s1. Figure S1: Variation in T2 comorbidities prevalence before and after specialist consultation.

## Abbreviations

The following abbreviations are used in this manuscript:

AD	Atopic dermatitis
AMS	Avoidance, modification, and slow eating
ARIA	Allergic rhinitis and its impact on asthma
CI	Confidence Interval
CRSwNP	Chronic rhinosinusitis with nasal polyps
EAACI	European Academy of Allergy and Clinical Immunology
EEsAI	Eosinophilic Esophagitis Symptom Activity Index
EGD	Esophagogastroduodenoscopy
ENT	Ear, nose and throat
EoE	Eosinophilic esophagitis
EREFS	EoE Endoscopic Reference Score
GINA	Global Initiative for Asthma
HPF	High power field
PPI	Proton pump inhibitors
VDQ	Visual Dysphagia Question
