# Supplementary figures and images for: The Overlooked Burden of Atopic Comorbidities in Eosinophilic Esophagitis: Insights from a Real-Life Comprehensive Multidisciplinary Evaluation
Source: J Clin Med. 2025 Oct 16;14(20):7322. doi: 10.3390/jcm14207322 (PMC12565518; doi:10.3390/jcm14207322)

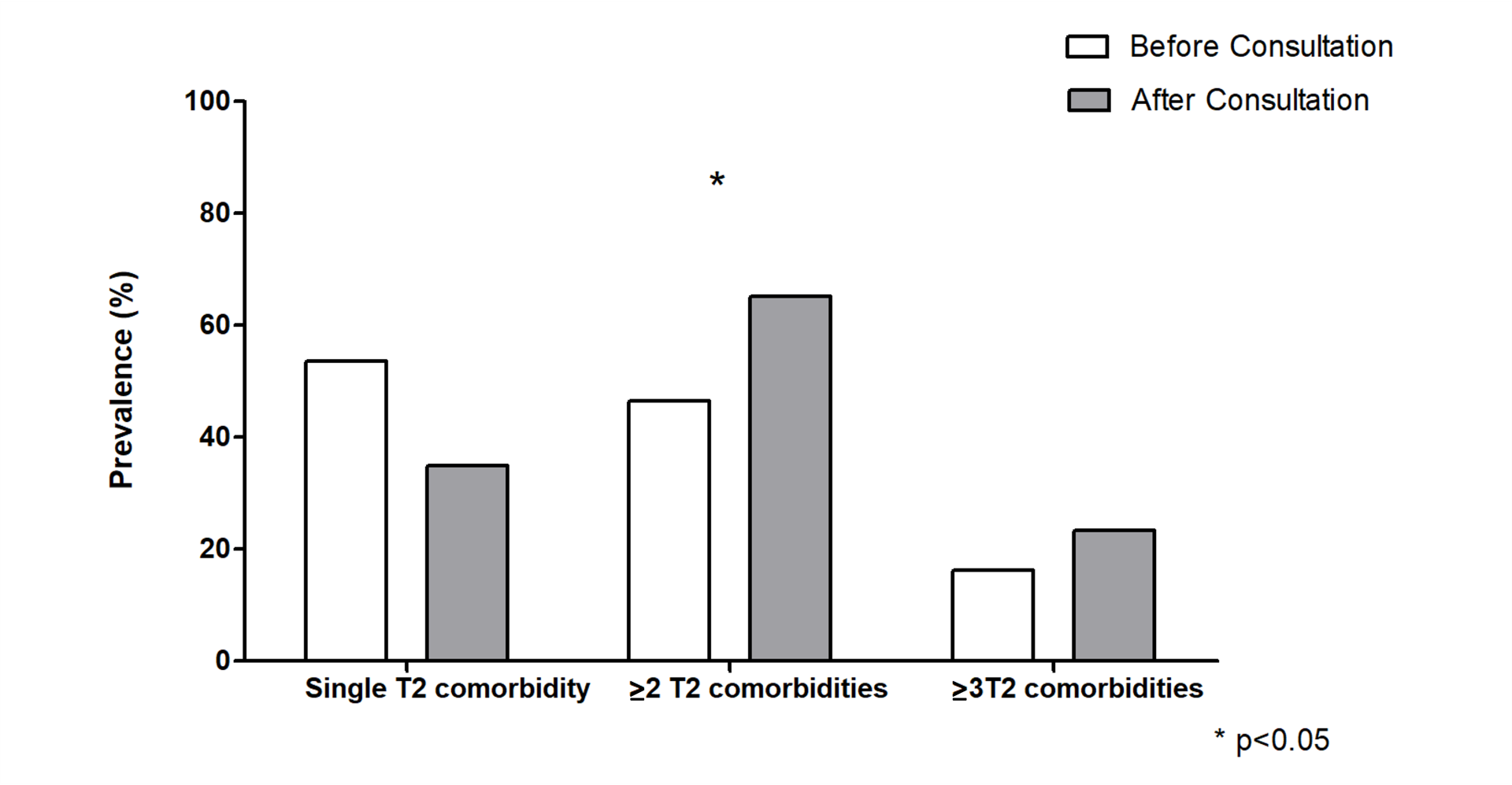

Supplement: Supplementary file 1 [file jcm-14-07322-s001.zip › jcm-3899561-supplementary.png]
